# Role of Paraoxonase 2 in Airway Epithelial Response to Oxidant Stress

**DOI:** 10.3390/antiox13111333

**Published:** 2024-10-31

**Authors:** Matthew S. McCravy, Zhonghui Yang, Jaime Cyphert-Daly, Zachary R. Healy, Aaron V. Vose, Haein R. Kim, Julia K. L. Walker, Robert M. Tighe, Heath G. Gasier, Jennifer L. Ingram, Loretta G. Que

**Affiliations:** 1 Division of Pulmonary, Allergy, and Critical Care Medicine, Department of Medicine, Duke University, Durham, NC 27710, USAjaime.cyphert@duke.edu (J.C.-D.); aaron.vose@duke.edu (A.V.V.); robert.tighe@duke.edu (R.M.T.); jennifer.ingram@duke.edu (J.L.I.);; 2 School of Nursing, Duke University, Durham, NC 27710, USA; 3 Department of Anesthesiology, Duke University, Durham, NC 27710, USA; heath.gasier@duke.edu

**Keywords:** asthma, ozone, paraoxonase, oxidant stress, mitochondria

## Abstract

Asthma is a widespread chronic lung disease characterized by airway inflammation and hyperresponsiveness. This airway inflammation is classified by either the presence (T2-high) or absence (T2-low) of high levels of eosinophils. Because most therapies for asthma target eosinophils and related pathways, treatment options for T2-low disease are limited. New pathophysiologic targets are needed. Oxidant stress is a common feature of T2-low disease. Airway epithelial expression of the antioxidant enzyme Paraoxonase 2 (PON2) is decreased in a well-recognized population of people with T2-low asthma and people with obesity and asthma. As a potential mechanism of increased oxidant stress, we measured the role of PON2 in lung oxidant responses using an environmentally relevant in vivo murine oxidant exposure (i.e., ozone) and in vitro studies with an immortalized human airway epithelial cell line BEAS-2B. Pon2-deficient (*Pon2^−/−^*) mice developed increased airway hyper-responsiveness compared to wild-type controls. Despite reduced alveolar macrophage influx, *Pon2^−/−^* mice exhibited increased nitrite production. In human airway epithelial cells incubated with hydrogen peroxide, PON2 knockdown (PON2KD) decreased mitochondrial function and inner mitochondrial membrane potential. These findings suggest that PON2 functions in defending against airway epithelial oxidant stress. Further studies are needed to elucidate the mechanisms linking PON2, oxidant stress, and asthma pathogenesis.

## 1. Introduction

Asthma is a chronic lung disease that affects people of all ages. In 2019, an estimated 262 million people were living with asthma globally, and 455,000 asthma-related deaths were reported [1]. Asthma is characterized by reversible airway obstruction, airway hyperresponsiveness (AHR), and chronic inflammation [2]. While the pathophysiology of asthma is heterogenous, asthma is clinically stratified broadly into two types of inflammation: type 2 (T2)-high and T2-low. The former is characterized by atopy and eosinophilic inflammation, whereas the latter is characterized by neutrophilic inflammation and elevated oxidant stress [3]. T2-high disease is well understood, and many therapies exist for people with poorly controlled T2-high asthma [4]. In contrast, the pathophysiology of T2-low disease is poorly understood, and targeted treatments for T2-low asthma are limited.

Several lines of evidence suggest that oxidant stress may be an important driver of disease in patients with T2-low asthma. Patients with T2-low asthma have higher levels of urinary 8-iso-prostaglandin F2a than patients with T2-high disease [5]. These levels positively correlate with asthma control and markers of airway remodeling, suggesting oxidant stress is part of the underlying pathology. Patients with comorbid obesity and asthma, a group typified by T2-low disease, have increased levels of 8-isoprostanes, a marker of lipid peroxidation, in exhaled breath condensate and lower nitric oxide bioavailability than lean people with asthma [6,7,8]. In addition, workplace-associated asthma is associated with increased airway oxidant stress and a T2-low phenotype [3,9,10]. While T2-low asthma is a heterogenous process, these findings suggest that airway oxidant stress is important in the development of T2-low asthma in a variety of settings.

Paraoxonase 2 (PON2) is an enzyme with antioxidant properties expressed in most human tissues [11]. Work in vascular biology and neurology has shown that PON2 is important in protecting endothelial cells from oxidant damage [12,13]. Data from the Human Protein Atlas shows that in healthy individuals, PON2 is highly expressed in the airway epithelium [14]. These combined observations suggest that PON2 may be important in defending airways against oxidant stress. However, little work has been completed on the function of PON2, specifically in the airway epithelium. When cellular *PON2* expression is decreased experimentally by silencing RNA (siRNA) in BEAS-2B cells, an immortalized human bronchial epithelial cell line, mitochondrial-derived reactive oxygen species (ROS) production is increased [15]. This finding suggests that loss of PON2 creates a predisposition towards enhanced oxidant stress and may be causative of inflammation. However, this postulate has not been investigated in vivo, and the physiological consequences are unknown. Furthermore, the mechanism whereby decreased PON2 affects mitochondrial function has not been studied.

We hypothesize that decreased PON2 results in worsened airway hyperresponsiveness, increased airway inflammation, and damaged mitochondria after an exogenous oxidant stressor. In the present study, we define a requirement for PON2 in lung responses to an oxidant challenge. We utilize ozone (O_3_) exposure as an environmentally relevant oxidant challenge that exhibits features of non-allergic asthma in rodent and human exposure studies [16,17]. We find that knockout of *Pon2* in mice leads to increased airway hyperresponsiveness following O_3_ exposure. This effect is associated with alveolar protein leak and airway nitrosative stress. Using human airway epithelial BEAS-2B cells, we find that *PON2* knockdown leads to inner mitochondrial membrane damage after an oxidant challenge. We conclude that PON2 is important in defending airway epithelium from oxidant stressors. While the effect of PON2 loss on macrophages and the vasculature has been known for some time, our study is the first report of PON2 loss effecting pulmonary mechanics and pulmonary epithelial function. This article is a revised and expanded version of an abstract entitled “Paraoxonase 2 Mitigates Mitochondrial Oxidative Stress in Airway Epithelial Cells”, which was presented at the American Thoracic Society International Meeting in San Diego, CA, USA, in May of 2024 [18].

## 2. Materials and Methods

### 2.1. Animals

C57BL/6J wild-type (WT) mice were purchased from the Jackson Laboratory (Bar Harbor, ME, USA). *Pon2*-deficient (*Pon2^−/−^)* mice on a C57BL/6J background were generously gifted from Dr. Srinivasa T Reddy of the University of California, Los Angeles, and backcrossed 10 generations on a C57BL/6J WT strain, then bred as a constitutive *Pon2^−/−^* line. This mouse line has been described previously [19]. In our experience, we did not observe any distinguishing physical characteristics of *Pon2^−/−^* mice compared to WT. Animal experiments were conducted in accordance with the American Association for the Accreditation of Laboratory Animal Care guidelines and approved by the Duke University Institutional Animal Care and Use Committee (IACUC Protocol number: A102-18-04, Animal Welfare number: D16-00123 [A3195-01]). 5–13 animals were used per group.

### 2.2. Ozone Exposure

To model T2-low asthma, mice were exposed to ozone, consistent with prior studies [20,21]. Male mice aged 6–8 weeks were exposed to filtered air (FA, control) or O_3_ (2 ppm) for 3 h [17]. In brief, mice were placed in stainless steel wire mesh cages within 55 L Hinners-style exposure chambers. Chamber air was maintained at 20–22 °C with 40–60% relative humidity and supplied at a rate of 20 changes/h. Ozone was generated by directing 100% oxygen through an ultraviolet light O_3_ generator. The concentration of O_3_ was continuously monitored with an O_3_ ultraviolet light photometer (Teledyne Instruments, model 400E, City of Industry, CA, USA). After exposures, mice were returned to their cages and allowed to recover for 24 h.

### 2.3. Airway Physiology Measurements

Airway responsiveness to nebulized 10–100 mg/mL acetyl-β-methylcholine (methacholine [MCh]; Sigma, A2251-25G) was measured 24 h following FA or O_3_ exposure [17]. Mice were anesthetized with urethane (1–2 g/kg; Sigma), tracheotomized and intubated with a metal endotracheal tube (18 G blunt needle) and placed on a 37 °C water-heated pad. Animals were mechanically ventilated at a rate of 150 breaths/min with a tidal volume of 10 mL/kg and a positive end-expiratory pressure (PEEP) of 3 cm H_2_O. To prevent spontaneous breathing, mice were given pancuronium bromide i.p. (0.8 mg/kg; Sigma-Aldrich, St. Louis, MO, USA) 5 min before measurements. For respiratory mechanics, 1 s single- and 3 s multi-frequency forced oscillation waveforms were applied using the Flexiware 7.6 software default mouse inhaled dose-response script (full script available upon request). The resulting pressure, volume, and flow signals were fitted to either a linear, single compartment model to obtain total respiratory system resistance (*R*_rs_) and elastance (*E*_rs_) or to a constant phase model to obtain Newtonian resistance (*R*_n_), tissue damping (*G*), and tissue elastance (*H*) [22,23]. Methacholine (MCh) was aerosolized using the FlexiVent (trademark) FX nebulizer attachment. The peak response at each dose was averaged and graphed with the average peak baseline measurement (the zero MCh dose using phosphate buffered saline [PBS] aerosol) for each group [24]. To characterize AHR, the provocative concentration of MCh that would cause a doubling of baseline resistance (PC100) for either Rn or Rrs was calculated [25]. In brief, natural log transformed Rn or Rrs dose-response data underwent linear regression to determine goodness of fit for PC100 analysis. Results having a goodness of fit (R^2^) greater than 0.50 were fit to the exponential growth function; log-linear interpolation was used to calculate PC100.

### 2.4. Bronchoalveolar Lavage (BAL)

After removal from the ventilator, lungs were washed 3 times with PBS through the tracheal cannula via gravity inflation to 20 cm H_2_O. BAL fluid was withdrawn with a syringe inserted into a side-port of the tubing and centrifuged to remove cells before concentrating twice using a centrifugal filter (Millipore, Burlington, MA, USA). BAL fluid cells were treated with red blood cell lysis buffer (BioLegend, San Diego, CA, USA, 420302) and resuspended in PBS. Total cell counts were determined using a Cellometer K2 (Nexcelom Biosciences, Rocky Hill, NJ, USA). Cell differentials were obtained by centrifuging a 100–150 µL aliquot of cell suspension onto slides using a Cytospin^TM^ 4 Cytocentrifuge (ThermoScientific). Slides were dried, stained with Hema 3^TM^ solution (Fisher Scientific, Hampton, NH, USA, 122-911), and viewed with light microscopy to quantify alveolar macrophages and leukocytes. Digital images of the cells in tagged image file (TIF) format were captured, and cells were counted in Adobe Photoshop in a blinded manner. An aliquot of BAL fluid supernatant was flash frozen and maintained at −80 °C for later measurement of cytokines using DuoSet enzyme-linked immunosorbent assays (ELISA) (R&D Systems, Minneapolis, MN, USA). Nitrate and nitrite levels were measured using a fluorometric nitrite/nitrate assay kit (Cayman Chemical Company, Ann Arbor, MI, USA, 780051).

### 2.5. Cell Culture

BEAS-2B cells, an immortalized human lung epithelial cell line, were purchased from American Type Culture Collection (ATCC^TM^, CRL-9609). A stable *PON2*KD line was generated by CRISPR-Cas9 targeted gene editing in consultation with the Duke Functional Genomics Shared Resource. BEAS-2B cells were either targeted at an Adeno Associated Virus Integration Site 1 (AAVS-1) locus as a control (knockdown at this site has no effect on cells) or targeted at the *PON2* locus with specific guide sequences. After verification at both transcription and translation levels, one targeted cell line with nearly 80% PON2 protein reduction was used in all cell culture experiments. BEAS-2B cells were grown to confluence in culture dishes coated with 1 µg/cm^2^ of PurCol^®^ (Advanced BioMatrix, Carlsbad, CA, USA 5005) using PneumaCult-ALI Basal Media (Stem Cell Technologies, Vancouver, BC, Canada, 05001). Hydrogen peroxide (H_2_O_2_, Sigma H1009) was diluted to working concentrations in cell culture media before experiments. Experimental cultures were then grown in media containing either 25, 50, or 100 µM of H_2_O_2_ for 24 h prior to mitochondrial assays. Control cultures were maintained in cell culture media. Response to H_2_O_2_ was assessed using 2′,7′ –dichlorofluorescin diacetate (DCF-DA, Abcam, Cambridge, United Kingdom, ab113851). Cells were incubated in 20 µM of DCF-DA for 45 min and then exposed to 100 µM H_2_O_2_ for 4 h, and then fluorescence intensity was measured in a plate reader.

### 2.6. Western Blot

Cells harvested from cell culture were sonicated in lysis buffer containing 20 mM Tris-HCl, pH 8.0, 0.5 mM Ethylenediaminetetraacetate (EDTA), 0.2 mM *Diethylenetriaminepentaacetate* (DTPA), 0.1% NP-40, and a protease inhibitor cocktail. Ten micrograms of cell lysates were separated on 4–20% precast polyacrylamide gels (BioRad Laboratories, Hercules, CA, USA) and transferred to polyvinylidene difluoride (PVDF) membranes. Membranes were blocked with PBS supplemented with 0.1% Triton X-100 and 3% milk for 1 h. Blots were incubated overnight with a 1:1000 dilution of anti-PON2 polyclonal antibody (Abcam, ab183710), washed in PBS + 0.1% Triton X-100 three times for 10 min each, then incubated with 1:10,000 horseradish peroxidase (HRP) conjugated anti-rabbit secondary antibody (R&D Systems, HAF008) for 30 min. After washing the membranes under the same conditions stated previously, signals were detected using the SuperSignal^TM^ West Pico PLUS Chemiluminescent Substrate and quantified by laser densitometry (ThermoFisher, 34578; BioRad Laboratories, Hercules, CA, USA).

### 2.7. Assessment of Mitochondrial Health

*PON2*KD and AAVS-1 control BEAS-2B cells (3 × 10^4^) were plated on a Seahorse XF96 collagen-coated plate (8 wells) for 24 h. Cells were washed twice with 200 µL of Dulbecco’s Modified Eagles Medium (DMEM, Aligent, Santa Clara, CA, USA) before measuring oxygen consumption rate (OCR) and extracellular acidification rate (ECAR) with Mito Stress Test (Agilent, Santa Clara, CA, USA 103015-100). The following concentrations were injected: 1 μM oligomycin (Oligo), 0.25 μM carbonyl cyanide, p-triflouromethoxyphenylhydrazone (FCCP), and 0.75 μM rotenone and antimycin A (R/A). To determine mitochondrial mass and mitochondrial membrane potential (ΔΨm) in BEAS-2B and AAVS-1 cells, 100 nM of MitoTracker^TM^ Green FM (ThermoFisher, M7514) and 50 nm of tetramethylrhodamine, methyl ester, and perchlorate (TMRM, ThermoFisher, I34361) were added to cells for 30 and 10 min, respectively. Cells were washed with 2 mL of cell culture media (PneumaCult, Stem Cell Technologies, Vancouver, Canada, 05001) containing penicillin, streptomycin, and amphotericin, and fresh media was added before imaging with a Keyence BX-800 fluorescence microscope using the 40× objective. Mitochondrial DNA (mtDNA) damage was assessed using DNA extraction (Qiagen, Hilden, Germany, 69504) and RT-PCR (Detroit R&D, Detroit, MI, USA, DD2H) kits per manufacturer’s instructions [26].

### 2.8. Statistics

Normality of continuous variables was assessed using the Shapiro-Wilk test. Airway physiology comparisons were made by one-way ANOVA. Rrs PC100 statistics for: all genotype/treatment groups met the criteria for normal distribution. The one-way ANOVA analysis showed a significant difference among the genotype treatment groups. An unpaired *t*-test with Welch correction factor for unequal variances demonstrated a significant difference between WT-O_3_ and *Pon2*^−/−^-O_3_ PC100. Rn PC100 statistics for: the *Pon2*^−/−^-O_3_ genotype/treatment group were the only non-normal distribution as assessed using the Shapiro-Wilk test. Kruskal–Wallis analysis showed a significant difference among the genotype treatment groups. An unpaired *t*-test showed no significant difference between WT-FA and WT-O_3_. A non-parametric Mann–Whitney demonstrated a significant difference between *Pon2*^−/−^-O_3_ and PON2-FA as well as WT-O_3_.

For cell culture experiments, all continuous variables were normally distributed and are presented as means with standard deviations. Hypothesis testing for means was performed by *t*-test or 2-way analysis of variance (ANOVA) with select pairwise comparison and Tukey’s Test to control for family-wise error rate. OCR and ECAR data were compared using multiple *t*-tests. False discovery rate correction by the Benjamini–Hochberg procedure with a value of <0.05 was considered significant.

## 3. Results

### 3.1. Ozone-Exposed Pon2^−/−^ Mice Have Increased Airway Hyperresponsiveness (AHR) Following Methacholine Challenge

**Figure 1:**
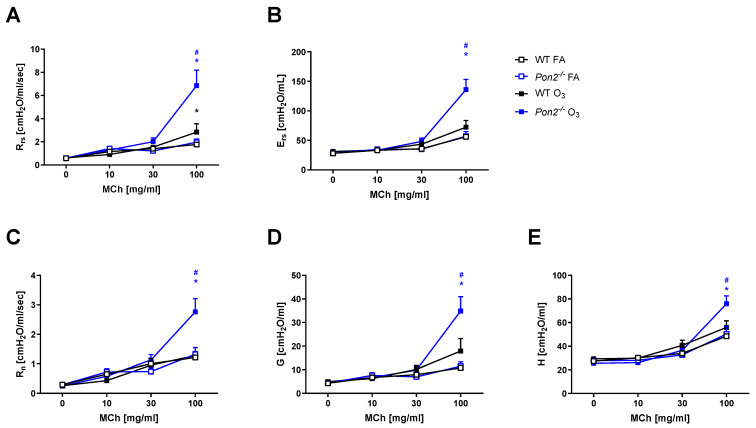
Airway mechanics measured in wildtype (WT) and *Pon2^−/−^* mice 24 h after a 3 h exposure to filtered air (FA) or 2 ppb ozone (O_3_). Airway responsiveness was determined by administering 0–100 mg/mL aerosolized acetyl-β-methylcholine (methacholine [MCh]). N = 12 for WT FA; N = 5 for *Pon2^−/−^* FA; N = 13 for WT O_3_; and N = 9 for *Pon2^−/−^* O_3_. (**A**) Rrs, airway resistance; (**B**) Ers, airway elastance; (**C**) Rn, Newtonian resistance; (**D**) G, tissue dampening; and (**E**) H, tissue elastance. Data presented as mean ± standard deviation. * *Pon2^−/−^* vs. FA control, *p* < 0.05. ^#^ *Pon2^−/−^* vs. WT, *p* < 0.05.

To determine the impact of PON2 in an environmentally relevant lung oxidant challenge, we exposed 6–8-week-old male C57BL/6 (WT, control) and *Pon2^−/−^* mice to FA or O_3_ (2 ppm) for 3 h. At 24 h post-exposure, we assessed airway mechanics following administration of increasing doses of aerosolized MCh. We did not observe differences in responses to MCh between WT and *Pon2^−/−^* mice following FA exposure (Figure 1, open boxes), nor did we find a statistically significant difference in PC100 between the two genotypes. Consistent with prior published studies [17], WT mice exposed to O_3_, when compared to FA-exposed WT mice, demonstrated elevated respiratory system resistance (Rrs), elastance (Ers), and tissue damping (G) (Figure 1, black open and closed boxes). Similarly, *Pon2^−/−^* mice challenged with O_3_, when compared to FA-exposed *Pon2^−/−^* mice, demonstrated elevated Rrs, Ers, and G (Figure 1, blue open and closed boxes). Compared to the O_3_-exposed WT mice, the O_3_-exposed *Pon2^−/−^* mice had exacerbated AHR with MCh (Figure 1, blue versus black closed boxes), as evidenced by worsened Rrs, Newtonian resistance (Rn), Ers and G, and elastance (H) (Figure 1).

**Figure 2:**
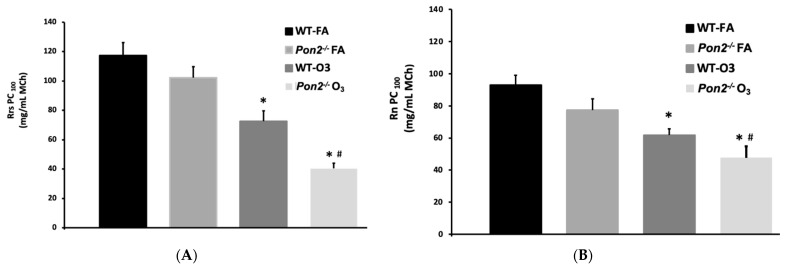
(**A**). PC100 Rrs Provocative concentration of methacholine (MCh) (PC100) that causes a doubling in Rrs from baseline (PC100). FA filtered air; O_3_ ozone; * *p* < 0.05 from FA; # *p* < 0.05 WT- O_3_ vs. *Pon2^−/−^* O_3_; one-way ANOVA with post-hoc *t*-test with Welch’s correction factor. (**B**). PC100 Rn. Provocative concentration of methacholine (MCh) (PC100) that causes a doubling in Rn from baseline (PC100). Data presented as mean +/− standard deviation FA filtered air; O_3_ ozone; * *p* < 0.05 from FA; # *p* < 0.05 WT-O_3_ vs. *Pon2^−/−^* O_3_ Kruskal–Wallis with post-hoc *t*-test with Mann–Whitney. N = 12 for WT FA; N = 5 for *Pon2^−/−^* FA; N = 13 for WT O_3_; and N = 9 for *Pon2^−/−^* O_3_.

Consistent with changes in Rrs discussed above, we observed no effect of genotype on PC100 in the FA-exposed groups (WT 117 ± 8.6, n = 12; *Pon2^−/−^* 102 ± 7.5, n = 5). O_3_ treatment caused PC100 to significantly decline in both WT (72.6 ± 7.0 n = 13) and *Pon2^−/−^* (40.4 ± 3.7, n = 9) mice, compared to their respective FA-treated control groups. However, the PC100 was significantly lower for O_3_-treated *Pon2^−/−^* mice than for similarly treated WT mice (Figure 2A). Consistent with changes in Rn discussed above, we observed no effect of genotype on PC100 in the FA-exposed groups (WT 92.9 ± 6.2, n = 12; *Pon2^−/−^* 77.4 ± 6.9, n = 5). O_3_ treatment caused PC100 to significantly decline in both WT (61.8 ± 4.0 n = 13) and *Pon2^−/−^* (47.7 ± 7.0, n = 9) mice, compared to their respective FA-treated control groups. However, the PC100 was significantly lower for O_3_-treated *Pon2^−/−^* mice than for similarly treated WT mice (Figure 2B). These data suggest that *Pon2* deficiency exacerbates O_3_-induced AHR.

### 3.2. Ozone-Exposed Pon2^−/−^ Mice Have Reduced Alveolar Macrophage Influx Despite Alveolar Damage and Increased Nitrate Production

**Figure 3:**
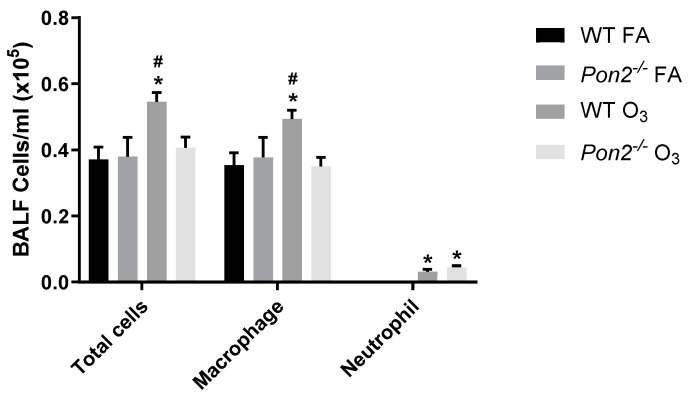
Total and differential cell counts in BAL fluid measured in *Pon2^−/−^* and WT mice 24 h following exposure to filtered air (FA) or 2 ppb ozone (O_3_). Cell counts were measured using a K2 Cellometer. Differential cell counts were performed by manual count on a single 40x image of a cytospin slide. N = 13 for WT FA; N = 5 for Pon2^−/−^ FA; N = 18 for WT O_3_; and N = 12 for *Pon2^−/−^* O_3_. Data presented as mean ± standard deviation. Comparison made by 2-way ANOVA. * *p* < 0.05 compared to FA. # *p* < 0.05 compared to the other genotype.

**Figure 4:**
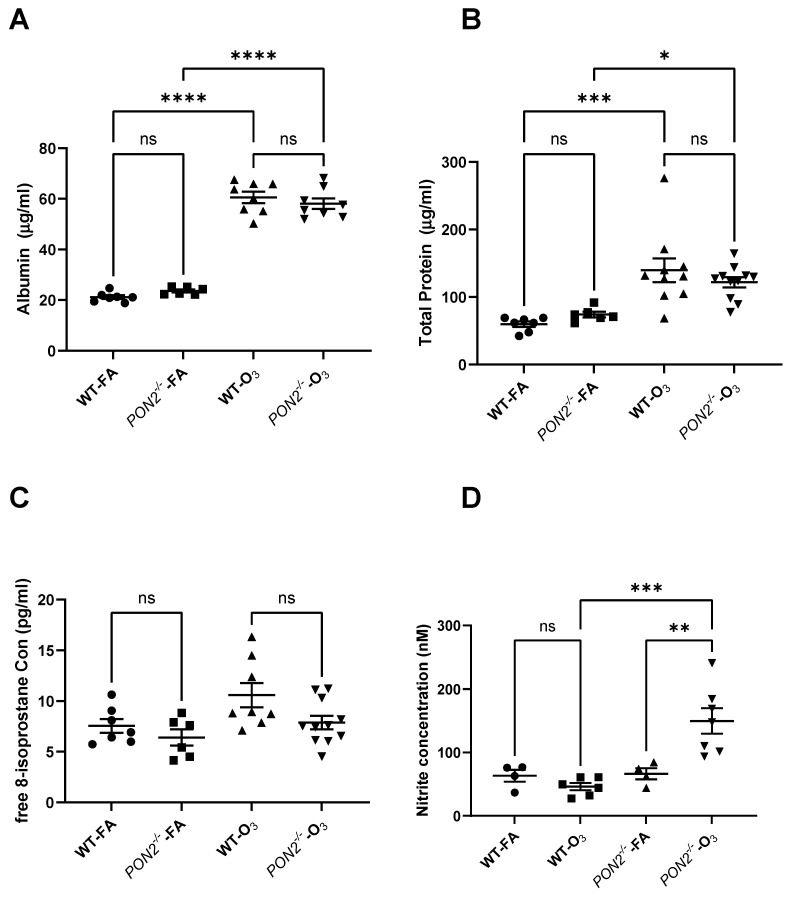
Airway permeability and oxidant stress markers in BAL fluid measured in *Pon2^−/−^* and WT mice 24 h following exposure to filtered air (FA) or 2 ppb ozone (O_3_). (**A**–**C**) Protein, albumin, and 8-isoprostane measured by ELISA. (**D**) Nitrite was measured by a fluorometric assay. N = 7 for WT FA; N = 6 for *Pon2^−/−^* FA; N = 10 for WT O_3_; and N = 11 for *Pon2^−/−^* O_3_ in the protein and albumin assays. N = 7 for WT FA; N = 6 for *Pon2^−/−^* FA; N = 8 for WT O_3_; and N = 11 for *Pon2^−/−^* O_3_ in the 8-isoprostane assay. N = 4 for WT FA; N = 6 for *Pon2^−/−^* FA; N = 4 for WT O_3_; and N = 7 for *Pon2^−/−^* O_3_ in the nitrite assay. Numbers differ due to availability of BAL fluid sample. Data presented as mean ± standard deviation. Comparison made by 2-way ANOVA. * = *p* < 0.05, ** = *p* < 0.01, *** = *p* < 0.001, **** = *p* < 0.0001, ns = not significant.

To define if *Pon2* deficiency also worsened O_3_-induced lung injury, we assessed BAL fluid for airspace inflammation as well as protein leak. BAL was performed 24 h post-FA or O_3_ exposure, and total cells and differentials were enumerated in BAL fluid. No differences were observed between WT and *Pon2^−/−^* mice exposed to FA (Figure 3). In WT mice, we observed increased total cells, macrophages, and neutrophils after O_3_ exposure, consistent with our prior research [17]. Alternatively, we observed no increase in total cells or macrophages in O_3_-exposed *Pon2^−/−^* mice, although an increase in neutrophils similar to that seen in WT mice was evident. We then measured total protein and albumin in BAL fluid as a measure of alveolar-capillary injury. In the FA exposure groups, we observed no difference in BAL protein and albumin between *Pon2^−/−^* and WT mice (Figure 4A,B). O_3_-exposed WT and *Pon2^−/−^* mice had increased BAL protein and albumin when compared to their respective FA groups. However, no difference was observed between O_3_-exposed WT and *Pon2^−/−^* mice, as they demonstrated similar O_3_-induced elevation in total protein and albumin. These data show that *Pon2*-deficient mice have reduced O_3_-induced alveolar macrophages despite comparable changes in airway permeability and alveolar injury. This finding suggests that inflammation cannot explain the increase in AHR seen in *Pon2^−/−^* mice following O_3_ exposure.

Direct measurement of reactive oxygen species is technically challenging in vivo due to the short half-lives of most ROS. To determine the effect of decreased PON2 expression on responses to an oxidant stress, we measured 8-isoprostanes and nitrite in BAL fluid in *Pon2^−/−^* mice exposed to FA or O_3_. 8-isoprostanes are a marker of lipid peroxidation, a downstream consequence of unchecked oxidant stress [27,28]. Nitrite results from ROS reacting with nitrogen at a cellular level and is also a reliable measure of prior oxidant injury [29]. We found no differences in 8-isoprostane levels between any group (Figure 4C). However, nitrite concentrations were higher in BAL fluid collected from O_3_-exposed *Pon2^−/−^* mice compared to both FA-exposed *Pon2^−/−^* mice and O_3_-exposed WT mice (Figure 4D). As nitrate is a downstream consequence of oxidant stress from environmental exposures, we reasoned that the increase in AHR may be due to increased oxidant stress at the level of the airway epithelial cell [30].

### 3.3. In Vitro Model of Airway Epithelial Oxidant Stress and PON2 Knockdown

**Figure 5:**
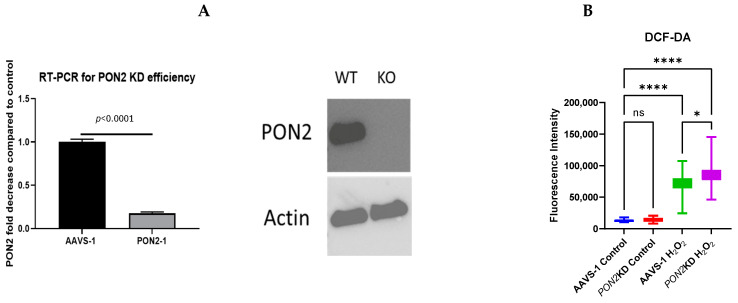
(**A**). CRISPR *PON2* knockdown. *PON2* was knocked down in BEAS-2b cells using CRISPR-Cas-9 with AAVS delivery (PON2-1). AAVS-1 cells were exposed to AAVS control. RT-PCR performed by normalizing to expression of the glyceraldehyde-3-phosphate (*GAPDH*) housekeeping gene. A significant decrease in *PON2* gene expression by RT-PCR (left) and PON2 protein expression by western blot (right) was observed. Hypothesis tested by two-tailed *t*-test. (**B**). DCF-DA. BEAS-2b cells were plated on a 96 well plate and stained with 200 nM of DCF-DA before being challenged with 100 μM of H_2_O_2_ for 4 h. (n = 4/condition). Data presented as mean ± standard deviation. ns = not significant * = *p* < 0.05, **** = *p* < 0.0001.

Because airway epithelial mitochondrial dysfunction has been linked to the pathogenesis of asthma and PON2 is localized to the inner mitochondrial membrane, we hypothesized that decreased PON2 production would impact airway epithelial mitochondrial function [15,31,32]. To define the mitochondrial implications of *PON2* deficiency, we generated an in vitro model of *PON2*KD using CRISPR-Cas9 targeted gene editing in BEAS-2B cells, resulting in an 80% reduction in *PON2* gene and protein expression (Figure 5A). We exposed *PON2*KD and control cells to H_2_O_2_ as an in vitro model of oxidant stress. We chose H_2_O_2_ because it is soluble in cell culture media and has been used previously to provoke oxidant stress in BEAS-2b cells [15]. The 100 µM dose was based on prior work investigating PON2 in BEAS-2b cells [15]. To confirm that H_2_O_2_ exposure provokes oxidant stress and to evaluate the effect of *PON2*KD, cells were stained with DCF-DA and exposed to H_2_O_2_ for 4 h, the maximum duration of DCF-DA efficacy. Quantification of DCF-DA staining demonstrated increased oxidant stress following H_2_O_2_ exposure in both *PON2*KD and AAVS-1 (control) cells, with the *PON2*KD cells exhibiting increased staining compared to control cells (Figure 5B). These findings suggest an important role for PON2 in epithelial cell defense against oxidant stress.

### 3.4. H_2_O_2_ Exposed PON2KD Airway Epithelial Cells Have Decreased Mitochondrial Function

**Figure 6:**
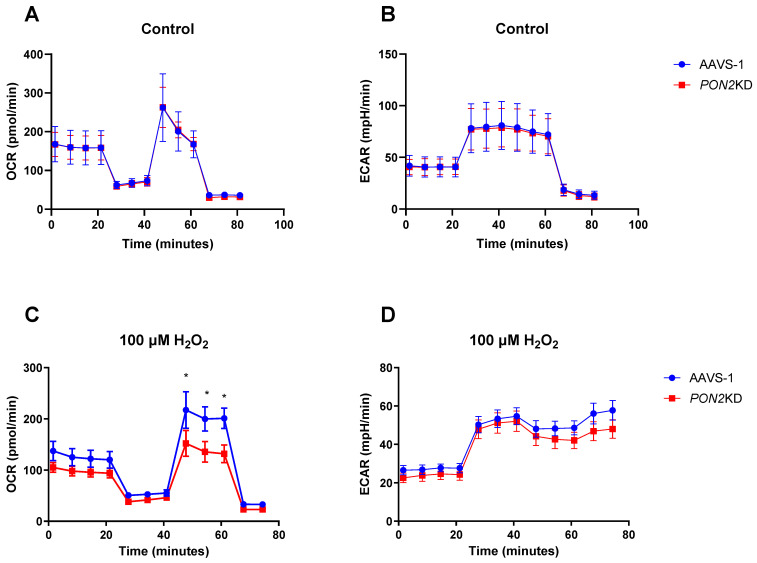
Mitochondrial Function. Cells were exposed to either 100 µM H_2_O_2_ or control media for 24 h. 10,000 cells/well were plated in Seahorse XFp plates (n = 3/conditions), and then mitochondrial function was assessed using a MitoStress Test Cartridge. Oxygen Consumption Rate (OCR) is a measure of total electron transport chain function. Extracellular Acidification Rate (ECAR) is a measure of glycolytic flux. (Top Row). No difference was observed in any parameter between the AAVS-1 cells and PON2KD cells under control conditions in both OCR (**A**) and ECAR (**B**). (Bottom Row) We observed a reduction in OCR (**C**) following FCCP addition in PON2KD cells, indicating a decrease in maximal respiration, without changes in baseline respiratory or electron leak. This finding indicates a decrease in mitochondrial space respiratory capacity (SRC). No differences were observed in any ECAR (**D**) parameter, indicating that changes in OCR were not due to an increase in glycolysis. Comparisons made by 2-way ANOVA with correction for multiple, repeated comparisons. Data presented as mean ± standard deviation * *p* < 0.05.

To determine the functional effect of PON2KD, we measured mitochondrial function using a Seahorse Bioanalyzer and a MitoStress test cartridge. Experimental cells were treated with 100 µM H_2_O_2_, consistent with prior published results, and control cells were treated with vehicle [15]. Under control conditions, basal respiration and maximal electron transport chain activity did not differ between AAVS-1 (control) and *PON2*KD cells (Figure 6A,B). In contrast, following the addition of 100 µM of H_2_O_2_ to the media of *PON2*KD cells, maximal OCR was reduced by 25%, reducing spare respiratory capacity (SRC, difference between basal and maximal OCR) by 40% (Figure 6C). In Figure 6D, we show that there is no change in ECAR, indicating no differences in glycolytic flux that could account for the change in mitochondrial activity. These data indicate PON2 is necessary to maintain mitochondrial function during heightened oxidant stress.

### 3.5. H_2_O_2_-Exposed PON2KD Airway Epithelial Cells Have Decreased Inner Mitochondrial Membrane Potential

**Figure 7:**
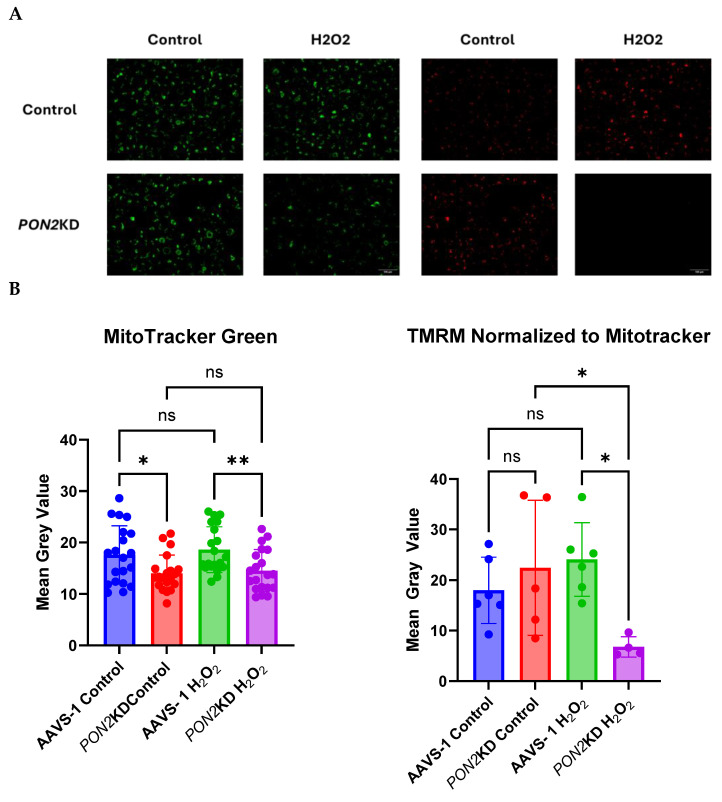
Mitochondrial function and mass in AAVS-1 and PON2KD cells. Cells were exposed to 100 µM H_2_O_2_ for 24 h. ΔΨm. PON2KD and AAVS-1 control BEAS-2b cells were grown to 70% confluence and stained with (**A**) MitoTracker Green (**Left**) and TMRM (**Right**) and imaged at 40x magnification. (**B**) Fluorescence intensity of Mitotracker Green (**Right**) and TMRM normalized to Mitotracker Green (**Left**). We observed a decrease in total mitochondrial mass in PON2KD cells compared to AAVS-1 control that does not change with H_2_O_2_ exposure. However, a decline in relative TMRM expression following H_2_O_2_ exposure in PON2KD cells compared to controls was noted, indicating a decrease in ΔΨ. Data presented as mean ± standard deviation. Comparisons made by 2-way ANOVA. ns = not significant, * *p* < 0.05, ** *p* < 0.01.

**Figure 8:**
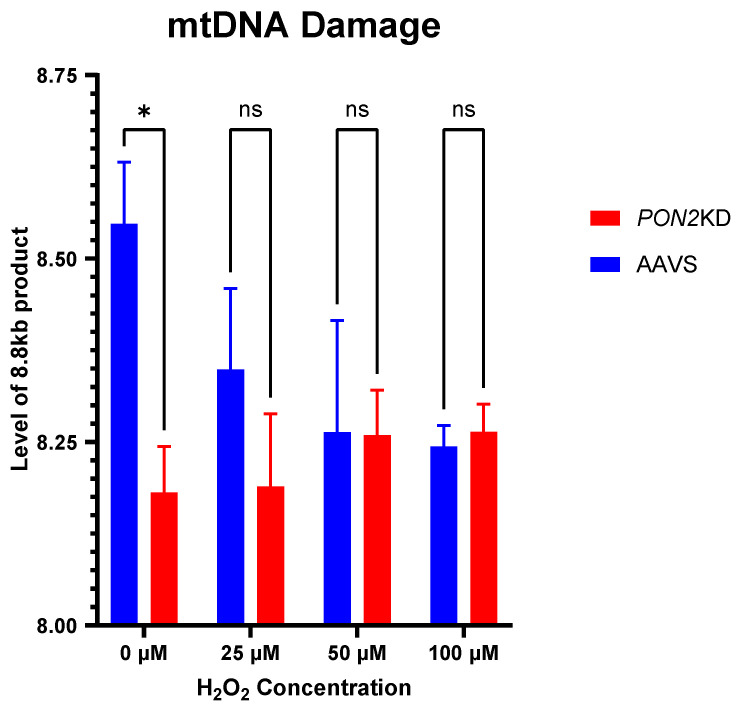
Mitochondrial DNA Lesions. BEAS-2b cells were exposed to 0, 25, 50, or 100 µM of H_2_O_2_ for 24 h. mtDNA lesions were measured by amplification of an 8.8 kB fragment and subsequent quantification by RT-PCR. N = 3/group. Data are depicted as means ± standard deviations. Comparisons made with multiple paired *t*-tests ns = not significant * *p* < 0.05.

To determine the mechanisms regulating altered mitochondrial function in *PON2*KD cells, we used Mitotracker Green and TMRM to measure mitochondrial mass and inner mitochondrial ΔΨm, respectively. Mitochondrial mass was lower in *PON2*KD cells compared to AAVS-1 controls (Figure 7A,B). In *PON2*KD cells, ΔΨm was reduced by 64% after a 24 h incubation with 100 µM H_2_O_2_ (Figure 7A,B). Mitochondrial DNA (mtDNA) is susceptible to damage and fragmentation following exposure to an oxidant challenge [33]. We reasoned that increased mtDNA fragmentation may explain the decrease in membrane potential following H_2_O_2_ challenge through reduced expression of electron transport chain subunits. To assess for mtDNA damage, we measured the relative amplification of a long (8.8 kb) mtDNA primer in exposed cells relative to controls. Increased amplification of the fragment corresponds to decreased mtDNA fragmentation (Figure 8). Under basal conditions, mtDNA damage was higher (0.6 kb) in *PON2*KD cells compared to AAVS-1 cells. The addition of increasing concentrations (25, 50, 100 μM) of H_2_O_2_ to the cells resulted in decreased 8.8 kb fragment amplification in WT AAVS-2 cells but no significant change in *PON2*KD cells. These data suggest that the oxidant-induced reduction in mitochondrial respiration in *PON2*KD cells is, in part, attributed to damage to the inner mitochondrial membrane versus mtDNA damage.

## 4. Discussion

In this study, we used a transgenic mouse model of non-allergic asthma as well as in vitro cell culture assays to demonstrate that PON2 is an important modulator of airway responses to oxidant stress. Prior work has shown that decreased *PON2* expression is associated with increased mitochondrial ROS [15]. We sought to define the effect on airway physiology and mitochondrial function of decreased PON2. Using *Pon2^−/−^* mice and acute ozone exposure as a model of non-allergic asthma, we show that decreased expression of *Pon2* results in more severe AHR. This increased AHR was accompanied by evidence of worsened cellular response to oxidant stress. Using *PON2*KD BEAS-2b cells, we show that decreased *PON2* leads to mitochondrial dysfunction in response to an oxidant stress. Together, our findings show that PON2 is an important defense against oxidant challenges in the airway.

Two prior studies have investigated the effect of PON2 in airway physiology, both focused on asthma [15,34]. The first is an epidemiologic study showing that a polymorphism in *PON2* was associated with increased asthma incidence [34]. Due to the nature of the study, the authors were unable to investigate any physiologic consequences of this observation. However, the second study used clinical samples from patients enrolled in other asthma studies, as well as immortalized human tissue lines, to demonstrate that airway epithelial *PON2* expression is decreased in participants with comorbid obesity and asthma and associated with increased mitochondrial ROS after an oxidant challenge. Our work expands on these findings by demonstrating that decreased expression of *Pon2* leads to worsened in vivo oxidant-induced AHR in mice, which is a defining physiologic feature of asthma. This finding suggests that decreased PON2 may be a mechanism driving respiratory symptoms in a subset of patients with asthma [2].

In addition to our airway physiologic observations, our findings show that PON2 maintains mitochondrial function following an oxidant stress. This observation is important because prior work has shown that decreased mitochondrial copy number, a surrogate of mitochondrial mass, is related to asthma incidence and severity, and vice versa [35,36]. Mitochondrial dysfunction is also necessary for the development of AHR in the ovalbumin model of allergic airway disease in mice [37,38]. However, it is not clear how mitochondrial dysfunction and airway mechanics are linked. Our work shows that decreased PON2 leads to both mitochondrial dysfunction in airway epithelial cells and AHR in a global *Pon2* knockout mouse model after oxidant stress. Because the airway epithelium is the primary defense against environmental oxidant stressors, decreased PON2 may be responsible for increased oxidants, inflammation, and AHR development [39].

Our work provides some insight into how mitochondrial health and AHR may be linked. We used O_3_ as a model of non-allergic asthma since it is a common environmental oxidant ubiquitous to all populations, induces AHR, and has been associated with exacerbations of several chronic lung diseases, including asthma [40,41]. It is well known that ozone provokes AHR. Our data show that loss of PON2 worsens AHR and that PON2 specifically defends against mitochondrial oxidant stress. We propose that primary loss of antioxidant defenses results in exaggerated responses to environmental triggers resulting in airway symptoms. This postulated scenario is of particular concern as climate change is associated with both ozone exposure and a variety of other environmental oxidants [42,43].

Some limitations of our study need to be considered. We were unable to use cultured primary airway epithelial cells from our mouse model to measure mitochondrial function. This deficit is due to technical limitations in measuring OCR in cells cultured at air-liquid interface. Future studies will address this limitation. Additionally, our mouse model is a global knockout, and we are not able to measure mitochondrial function in vivo, limiting our ability to draw firm conclusions on the specific connection between airway epithelial mitochondrial function and AHR. However, several technologies are available to address these concerns; our future studies will make use of both tissue-specific knockout and mitochondria reporter mice to narrow down the role of PON2 in airway epithelium barrier function and AHR and connect these effects to mitochondrial function in vivo. Similarly, our model of pure oxidant stress cannot replicate the full complexity of T2-low asthma. However, by isolating these effects, we can still shed light on this aspect of the disease process.

In conclusion, our study shows that decreased expression of *PON2* leads to worsened AHR and increased nitrite production after exposure to O_3_ in vivo and mitochondrial dysfunction in airway epithelial cells in vitro. These observations are important as AHR is a characteristic feature of asthma and mitochondrial dysfunction is associated with increased asthma severity [32]. Our findings suggest that efforts to boost mitochondrial antioxidant defense and *PON2* expression in patients with T2-low asthma may be beneficial and are worthy of further research.

## Data Availability

The original contributions presented in the study are included in the article, further inquiries can be directed to the corresponding author.
